# A novel cross-institutional college internship program to train future diverse leaders in clinical research with data-driven approaches to assess impact

**DOI:** 10.3389/fphar.2023.1294535

**Published:** 2023-12-06

**Authors:** Julia Derk, Kafui Dzirasa, Tracie Locklear

**Affiliations:** ^1^ The Collective for Psychiatric Engineering at Duke University School of Medicine, Durham, NC, United States; ^2^ Duke-North Carolina Central University Clinical and Translational Science Institute Workforce Development Core, Durham, NC, United States; ^3^ Howard Hughes Medical Institute, Durham, NC, United States; ^4^ Department of Psychiatry, School of Medicine, Duke University, Durham, NC, United States; ^5^ Biomanufacturing Research Institute and Technology Enterprise, North Carolina Central University, Durham, NC, United States

**Keywords:** clinical research, internships, cross-institution collaboration, diversity equity and inclusion, key competence in science and technology

## Abstract

The field of Clinical Research, like many other scientific disciplines, has struggled to recruit and retain talented researchers from diverse communities. While there is a strong history of documenting the problem, having a diverse and inclusive workforce is hindered by the lack of data-driven approaches, cross-institutional partnerships, access to mentors, and positive immersive experiences for people from underrepresented groups. Here, we describe a novel initiative for North Carolina Central University Clinical Research Sciences Program (NCCU-CRSP) student interns to partner with Duke University to have immersive clinical and pre-clinical research training in a 15-week internship as the culminating experience towards their degree for a Bachelor of Science in Clinical Research. The goals of the internship are: 1) to give hands-on training to enhance the impact of classroom-based learning, 2) broaden their understanding of the wide swath of positions available to them, 3) promote their sense of self-efficacy, confidence, science identity, research identity, and connections to the pre-clinical and clinical community, and 4) prepare them to be workforce ready upon graduating. The students dedicate 75% of their time to clinical research with Duke University at Pickett Road and 25% to pre-clinical research in the Collective for Psychiatric Neuroengineering in the Duke Psychiatry Department of the School of Medicine. They will also receive eight 1-h professional development training sessions from the Duke-NCCU Clinical and Translational Science Initiative’s Workforce Development Team and five 1-h sessions based on the Entering Research Curriculum developed by the Center for the Improvement of Mentored Experiences in Research (CIMER). Finally, they will be brought in as a cohort and coached on peer mentoring and mutual support frameworks to enhance their sense of community. These student-interns will perform pre- and post-internship self-assessment surveys to quantify their self-efficacy, feelings of belonging, access to research opportunities and mentors, and to give details of their future education and career goals. We will evaluate the impact of the internship using validated tools and apply these findings for future optimization of program design and tactical advice for other programs with shared missions. Furthermore, we will email them on an annual basis with follow-up surveys to assess the longitudinal impact of this internship program, their educational experiences at NCCU, what job titles they hold, how prepared they feel for their roles, and what they hope their future career trajectory will be. Collectively, these approaches will apply theoretical frameworks developed by social and cognitive psychology, vocational theory, and educational research to clinical research training with the goals of recruiting and training talented and diverse leaders within clinical research. We hope that by evaluating our successes, failures, strengths, and liabilities through empirically derived evidence we will also inspire future studies that use data-driven approaches to elevate our approaches as we work together to train and recruit talented researchers from diverse communities into our scientific enterprise and to launch them with more in-depth experiential learning that will empower them to succeed.

## Introduction

The field of clinical research has identified an immense need to develop a more diverse and inclusive workforce (Locklear et al.; NSF, 2022). While Black, African American, Latino/a, Hispanic, Indigenous, and Native individuals comprise more than 30% of the US population, they comprise less than 19% of bachelor’s degrees and 15% of PhDs in biological sciences, thereby designating them as Historically Underrepresented (HU) groups (Statistics, 1994; NSF, 2022). Furthermore, the significant lack of diverse representation amongst clinical trial participants can drive a slew of problems, including compromising the generalizability of clinical research findings, undermining trust in the medical establishment and research community, and compounding health disparities (Improving Representation, 2022). There are ample emerging data illuminating how important physician-patient race concordance is, particularly in the context of boosting health service utilization and reducing infant mortality rates (LaVeist et al., 2003; Alsan et al., 2018; Greenwood et al., 2020). Similarly, studies have shown that more diverse personnel at clinical trial workplaces correlates to increased diversity in patients recruited to studies and African American women report to be more motivated to join studies if there is race concordance with the practitioners of the study (Frierson et al., 2019; Tufts Center for the Study of Drug Development, 2021). While many have identified the issues of a lack of diversity in the clinical research workforce and have measured its scope, novel and innovative tactics are required if we want to make real change (Byars-Winston et al., 2011; Valantine and Collins, 2015; Byars-Winston et al., 2016; Hitchcock et al., 2017; Winn Ariel et al., 2019). The number of clinical trials and the scope of clinical research are rapidly expanding, which requires an influx of workforce-ready individuals into our field if we aim to keep up with demand (US National Library of Medicine). We need education, training, and workforce development approaches paired with high-quality assessments to quantify the impact of interventions on 1) diversifying our field and 2) increasing the number of workforce-ready applicants to meet the ever-expanding needs of the field. Our methods must overcome the long-standing legacies of systemic inequality and structural racism to improve access to the education, training, and mentoring required for a robust career in clinical research. The time has come for large-scale investments to build sustainable, multifaceted, and empowered training programs that will equip the next-generation of diverse leaders in clinical science to achieve their goals and contribute to transformative breakthroughs.

We are fortunate to live in a time where there is tremendous innovation around how to evaluate the impact of educational interventions to improve diversity, equity, and inclusion outcomes. Specifically, six key domains have been empirically shown to improve retaining diverse individuals in the research enterprise: 1) development of scientific identity, 2) development of research identity, 3) increased self-efficacy, 4) improved sense of belonging, 5) expanding expected outcomes, and 6) good mentorship (Bakken et al., 2006; Graham et al., 2013; Byars-Winston et al., 2016; Winn Ariel et al., 2019). In this perspective article, we detail a pioneering internship program at the interface of Duke University clinical and pre-clinical research and North Carolina Central University (NCCU), a predominantly Black university located five miles away from Duke. 95% of NCCU students come from underrepresented racial and ethnic backgrounds and 61% of students were awarded Pell Grants last year, thus this group of students is a significantly more diverse population of individuals than the current population of clinical research professionals. Furthermore, we describe the implementation of validated tools to assess the impact of the internship on the six key domains described above that have been previously shown to promote recruitment and retention into any field. Through this pilot project, we hope to build long-term sustainability for our student interns and to contribute to a moonshot goal of diversifying the clinical research workforce to reflect the US population demographics by 2030 through data-driven approaches that promote equity and inclusion of talented, diverse leaders within the field of clinical research (Envisioning a TransformedBoard on Health Sciences PolicyHealth and Medicine DivisionNational Academies of Sciences et al., 2021).

## Program design and methodology

This perspective article presents a novel approach for a capstone internship experience for seniors graduating with a Bachelor of Science in Clinical Research from NCCU. We apply adapted tools to assess the impact this experience has on the six key domains that have been shown to improve recruitment and retention in communities and vocational tracks for other fields. The intern participants will gain multifaceted, cross-institutional experiences in which they dedicate 75% of their time to clinical research with Duke University at Pickett Road paired with a Clinical Research Coordinator doing an NIH funded study. The other 25% of their time will be devoted to conducting pre-clinical research in the Collective for Psychiatric Neuroengineering focused on genetic engineering and gene therapy principles. In both settings, they will conduct rigorous analyses of the literature, develop technical skills, and work collaboratively to achieve progress in their respective studies. They will also receive eight 1-h sessions of professional development training from the Duke-NCCU Clinical and Translational Science Initiative’s Workforce Development Team and five 1-h sessions based on the Entering Research Curriculum developed by the Center for the Improvement of Mentored Experiences (Balster et al., 2010). The student interns will be brought in as a cohort and coached on peer mentoring and mutual support frameworks to enhance their sense of community. There will also be a network of formal and informal mentors who are specifically dedicated to improving their technical and interpersonal skills for success in the clinical research workforce.

Traditionally, there are no required educational backgrounds or specific competencies to become a clinical research professional. However, there are still stark diversity and equity issues in the field. There is mounting pressure to grow the clinical research workforce as the number of registered trials increases. However, strictly adding more people won’t solve all the field’s issues. If we aim to promote equity to reduce health disparities, we must maintain a steadfast focus on the goals: 1) growing this workforce, 2) recruiting talent from diverse communities, and 3) enhancing feelings of belonging and inclusion to retain individuals from diverse backgrounds throughout the process. The NCCU Clinical Research Science Program aims to create a robust curriculum that trains students from diverse backgrounds to develop core competencies that will empower them to be leaders in clinical research. In addition to their classroom training in pursuit of a Bachelor of Science, this internship program aims to facilitate them gaining professional skills, confidence, self-efficacy, sense of belonging, and to promote their identity as top-tier researchers and scientists. We hope to empower a well-qualified cohort of students to obtain roles as clinical research professionals with strong skills in core competency domains that the Joint Task Force for Clinical Trial Competency and Clinical Research Professional Workforce Development has recently developed (Sonstein and Jones, 2018).

While we have trained to the competencies outlined in the article above, the learning objectives for the internship are to test the “workability” of our cohort, specifically as follows.

1) Be able to follow Good Clinical Practices in the conduct of clinical research
2) Understand and describe the roles of various clinical research professionals within the clinical research team
3) Apply professionalism and interpersonal skills to ensure success in clinical and scientific workplaces
4) Build skillsets in quantitative research
5) Apply literature review skills to develop and share new knowledge in clinical and scientific research workplace
6) Execute well-researched presentations with confidence
7) Incorporate professional and research experiences into resumes and increase marketability of the scholars

Importantly, we will administer in-depth pre- and post-evaluations that rely on validated tools to determine the impact of our intervention on promoting recruitment and inclusion in a field (Gloria and Kurpius, 2001; Hurtado et al., 2007; Byars-Winston et al., 2011; Trujillo and Tanner, 2014; Byars-Winston et al., 2016). From these self-assessment surveys, we will quantify their changes in self-efficacy, feelings of belonging, access to research opportunities, access to research mentors, detail their future career goals, and use qualitative and quantitative measures to assess their confidence, comfort, and expectations of success within the clinical research field (Table 1). By doing so before and after the internship experiences of multiple cohorts, we will develop a robust understanding of the impact of this internship experience on our student interns with depth and nuance based on empirical evidence. Additionally, we will query the internship preceptors for each student to evaluate the interns’ skills, growth, and readiness to enter the workforce. Finally, we will do an annual survey following up with each intern to determine their career trajectory longitudinally and to ask how ready they felt to take on their new positions, to climb the career ladder in Clinical Research, and to determine if they were retained long-term in the field.

**TABLE 1:** Variables to be evaluated through pre- and post-assessments of student interns in the NCCU Clinical Research Science Program.

Self-efficacy
Confidence in conducting high quality clinical research
Confidence in understanding how a biology laboratory operates
Confidence in molecular biology techniques in the laboratory
Confidence in understanding and describing the roles of various clinical research professionals within a clinical research team
Confidence with applying good clinical practices to clinical research
Confidence in your capacity to succeed in clinical or pre-clinical research
Confidence in yourself professionally, in general
Confidence in talking about clinical research with other professionals
Confidence in talking about pre-clinical research with other professionals
Confidence in applying statistics to a research question
Confidence conducting quantitative research
Confidence conducting a literature review
Confidence applying a literature review to share knowledge with others in the workplace
Confidence in executing a well-researched presentation
Confidence in applying to your first job after you graduate
Confidence in having a strong career in clinical research that supports your goals in life

Sense of belonging
Comfort level in a scientific lab
Comfort level in a clinical research setting
Comfort level in any professional work setting
Satisfaction with access to clinical research opportunities
Satisfaction with access to clinical research mentors
Access to opportunities and mentors in clinical research.

Expected outcomes
A clinical research career would allow me to work that makes a difference in people's lives or society.
A clinical research career would allow me to work that I find satisfying
A clinical research career would allow me to go into a field with high employment demand
A clinical research career would allow me to get respect from other people
A clinical research career would allow me to earn an attractive salary.
How important do you think it is to have clinical research mentors?
How important do you think internships are to your Confidence in yourself?
How important do you think it is to have access to role models?
How important do you think it is to have peer colleagues in your STEMM career?

Clinical research Identity
Current Major
Future plans for higher education
Future plans for job titles to hold
I can picture myself being successful as a clinical science researcher
I am already successful as a clinical science researcher

Access to mentors and experiences
Clinical research scientist mentor numbers and frequency of contact
Hands-on research experience in clinical research opportunities
Pre-clinical research scientists mentor numbers and frequency of contact
Clinical research or pre-clinical research workplace access
Pre-clinical research science mentor numbers and frequency of contact
Clinical or pre-clinical research internship access

**FIGURE 1:**
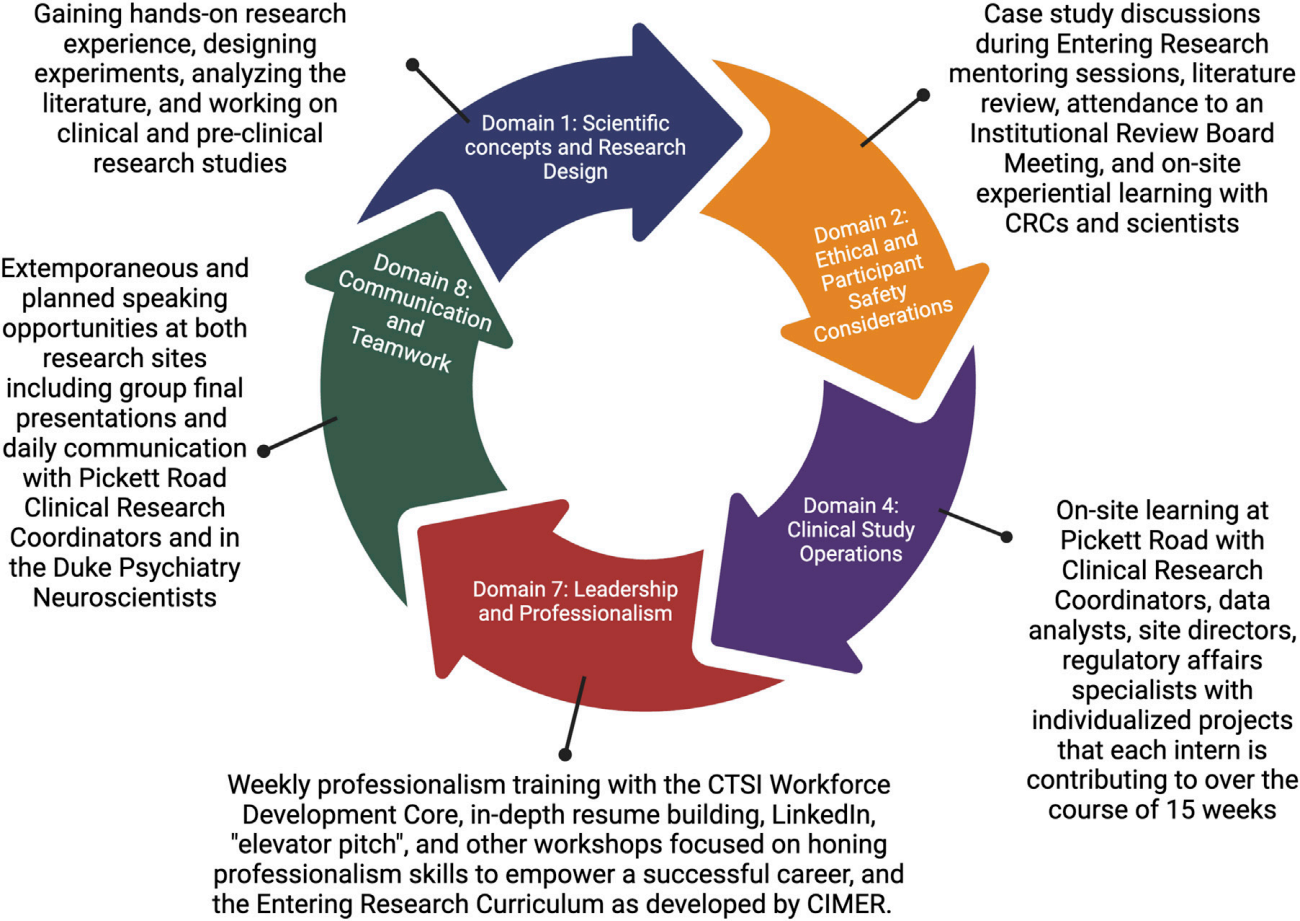
Joint Task Force key competency domains for clinical research and their alignment with internship activities.

Many of these key focus areas overlap with the goals of the Joint Task Force for Clinical Competencies Domains (Figure 1) (Sonstein and Jones, 2018). We will develop their skills for Competency 1: Scientific Concepts and Research Design in both the Clinical Research at Pickett Road and the Pre-Clinical Molecular Neuroscience Research with the Collective for Psychiatric Neuroengineering by giving hands-on research experiences, helping interns to design experiences, analyze the literature, develop technical and interpersonal expertise to accomplish the goals of their research, and to take pieces of the studies from conception to execution and analysis. They will have thorough Ethical Training which aligns with Domain 2, including engaging in the Entering Research Curriculum (focusing on case studies of responsible conduct in research, setting expectations, and ethics discussions), attending an IRB meeting, and online learning with Clinical Research Coordinators and Experimental Scientists daily. We are giving them hands-on training to apply what they’ve learned in the classroom regarding Competency 4: Good Clinical Practice and Operations in the Clinical Research Setting. By promoting self-efficacy, a sense of belonging, and scientific identity in our students, we empower them to develop Competency 7: Leadership and Professionalism and Competency 8: Communication and Teamwork. We are further supporting Competency 8 through immersive experiences where they must work as a team to solve problems, give presentations, and develop their collaboration skills to accomplish the assigned tasks as an internship cohort. Taken together, we aim to integrate the advice and direction from Clinical Research and Research Mentorship experts to provide a holistic internship experience that will empower our students to be workforce-ready by up-skilling them in the key domains identified by this highly respected council.

## Discussion and future remarks

Our mission is to develop a clinical research training program that diversifies the field through a robust understanding of the strengths and liabilities of our approach over multiple cohorts. This depth of understanding will be mission-critical to maximize our capacity to empower our student interns to become leaders in clinical research. We aim to apply the powerful tools and foundational theoretical frameworks that leaders in vocational psychology have developed in response to the critical need to increase the number of workforce-ready clinical researchers while concurrently diversifying the workforce. Analyzing our impact along the way will allow for iterative design to optimize our program in the future. We will use the gained knowledge to generate tactical advice for other programs with shared missions for the mutual edification of our programs.

Additionally, we will follow our interns longitudinally throughout their career trajectories to allow us to develop systems to assess the long-term impact interventions have on retention. In the near future, we also aim to query future employers as to how “job-ready” the graduates from our program were compared to other competitive applicants and what their primary foci are in the hiring process into their field. Finally, we will continue to document this journey and raise funds for this training program to bring about awareness and promote sustainability that will allow for a larger and more meaningful impact on future cohorts and larger participant pools.

While these strategies may only provide a partial solution to the long-standing systems of inequality that we must overcome, they provide three key improvements: 1) a framework that can be utilized and repurposed as we collectively work to recruit, train, and retain diverse researchers in clinical research (or other fields), 2) the application of vocational psychology theoretical frameworks to clinical research training opportunities as a novel synergistic integration of the fields, and 3) an opportunity to continue to revise our training models based on data-driven insights. If successful, we will contribute to the intentional deconstruction of systemic barriers that have historically driven health, economic, and educational inequalities that have held us back as a society and as a clinical research field.

## Data Availability

The original contributions presented in the study are included in the article/Supplementary material, further inquiries can be directed to the corresponding authors.
